# Icariin Ameliorates Cyclophosphamide-Induced Renal Encephalopathy by Modulating the NF-κB and Keap1-Nrf2 Signaling Pathways

**DOI:** 10.3390/ijms26104838

**Published:** 2025-05-19

**Authors:** Meiling Shi, Hong Kan, Yijia Tang, Lanshi Tian, Xiangjuan Guo, Weijia Chen, Jianan Geng, Ying Zong, Yunfeng Bi, Zhongmei He

**Affiliations:** 1 College of Chinese Medicinal Materials, Jilin Agricultural University, Changchun 130118, China; anxia143341@163.com (M.S.); khdyx681@163.com (H.K.); 18205037129@163.com (L.T.); gxj20002024@163.com (X.G.); chenweijia_jlau@163.com (W.C.); gengjianan@jlau.edu.cn (J.G.); zongying@jlau.edu.cn (Y.Z.); 2 School of Life Sciences, Jilin University, Changchun 130118, China; tangyj1323@mails.jlu.edu.cn; 3 College of Food Science and Engineering, Jilin Agricultural University, Changchun 130118, China

**Keywords:** icariin, cyclophosphamide, inflammation, apoptosis, serotonin, cognitive impairment

## Abstract

Chemotherapy-induced renal encephalopathy (RE) is a disease characterized by cognitive impairment of the brain caused by impaired kidney function for which there is no definitive treatment. Icariin (ICA), the main active component of *Epimedium*, has a good nervous system protection and anti-neuroinflammation effect, but its effect on the brain injury caused by renal insufficiency as a result of chemotherapy remains unclear. In this study, we demonstrated that 100 mg/kg ICA can not only successfully interface with serotonin and regulate hormone levels but also ameliorates kidney damage and cognitive impairment in cyclophosphamide (CTX)-induced RE mouse models and inhibits inflammation, oxidation, and apoptosis by regulating NF-κB, keap1-Nrf2, and apoptosis pathways. In order to further study the protective effect of ICA on RE, we used CTX-induced HT22 and HEK293 cell injury models, and the ICA intervention showed that ICA could prevent apoptosis by regulating the expression of the apoptosis-related proteins caspase-3, Bcl-2, Bax and BDNF. Overall, our study provides a basis for further investigation of the therapeutic potential of ICA in the treatment of neurodegenerative diseases in the context of renal dysfunction, and further studies are needed at a later stage to fully elucidate the underlying molecular mechanisms.

## 1. Introduction

The use of chemotherapy drugs and oxidative damage can lead to brain damage, which can cause cognitive dysfunction. Brain injury is the leading cause of death and disability worldwide, with studies reporting that 150–200 people per million under the age of 40 become disabled each year [1]. The lesions include apoptosis, necrosis, neuroinflammation, and oxidative stress, and the diffuse inflammatory infiltrate results in behavioral deficits caused by the necrosis of neuronal cells, which is now an essential source of cognitive dysfunctions [2].

Cyclophosphamide (CTX) is an alkylating agent in different cancer chemotherapies [3]. CTX has been found to stabilize hematology and treat prostate cancer [4], breast cancer [5], and neuroblastoma [6] and counteract immunosuppression in cancer [7]. Long-term chemotherapy can cause toxicity to various organs [8], with up to 35% to 60% showing persistent cognitive impairment (CRCI), 50–90% developing cognitive dysfunction, and 2–5% developing Alzheimer’s disease (AD) [9,10]. It has been suggested that renal pathology may be an essential factor in the development of associated cognitive impairment [11]. Some studies have found that an increased activity of brain-derived neurotrophic factor (BDNF) in the hippocampus plays a role in tonifying the kidney and brain, calming the mind, and relieving depression [12]. Studies have confirmed that the neurons innervating the kidney are symmetrically distributed in the midbrain and posterior brain, indicating that the kidneys and brain are inter-related [13]. Studies have shown that patients with renal cell carcinoma can exhibit mild cognitive impairment and dementia [14]. *Epimedium* is known for its anti-inflammatory and antioxidant properties, which can fight the progression of CNS diseases, such as neurodegenerative diseases [15], and ICA has shown potential preventive and therapeutic effects in the nervous system [16].

Serum sex hormones can regulate physiological functions and connect the brain and kidneys [17]. The follicle-stimulating hormone (FSH) and luteinizing hormone (LH) secreted by the pituitary gland promote gonadal development, while testosterone (T) is a steroid hormone produced by a person’s testicles and adrenal glands [18]. 5-Hydroxytryptamine (5-HT) is capable of ameliorating the abnormal expression of inflammation and apoptosis-related proteins, and dopamine (DA) can also enhance the excretion of sodium to regulate renal tubule function and minimize the occurrence of kidney injury [19]. Serotonin is involved in a variety of cognitive functions including memory and can improve spatial recognition memory and spatial cognitive memory deficits in mice. However, current research on the pathological mechanism of renal encephalopathy mainly focuses on inflammatory factors, and research on 5-HT levels and neurotransmitter dysfunction is still insufficient [20]. Molecular docking technology enables us to explore effective compounds with predetermined targets in modern drug discovery processes for further prediction and validation [21].

Oxidative stress is one of the factors causing brain injury, which is the result of an imbalance in the ratio of intracellular pro-oxidants and antioxidants [22], and the accumulation of reactive oxygen species (ROS) exacerbates brain injury [23], damages organelles, and induces apoptosis in brain cells [24]. ROS are considered essential activators of NOD-like receptor thermal protein domain associated protein 3 (NLRP3), and the NLRP3 inflammasome pathway is central to initiating and maintaining neuroinflammation [25]. Apoptosis is one of the cellular mechanisms of brain injury, and the mouse hippocampal neuronal cell line HT22 can be assessed for neuronal injury [26]. BDNF and neurotrophic protein 3 (Ntf3) have a protective effect on neurons, and related studies have shown that curcumin may protect HT22 cells from acrolein by modulating the BDNF/TrkB signaling pathway [27]. It was found that ICA alleviated glial cell-mediated neuroinflammation and induced DA neuroprotection in an NRF2-dependent manner [28].

There is bidirectional regulation between BDNF and inflammation, and elevated levels of inflammatory mediators lead to a decreased expression of BDNF, which plays a vital role in negatively regulating inflammation in the brain [29]. Tumor necrosis factor-α (TNF-α) in the nervous system is mainly secreted by glial cells, and its expression is significantly elevated after focal brain injury [30]. The hippocampus is located in the medial temporal lobe of the limbic brain, a vital part of the brain involved in the formation and storage of declarative spatial memories, governing spatial learning and memory deficits. Ginsenoside Rg3 enhanced neurological and motor functions in mice after TBI, attenuating hippocampal neuronal neuroinflammation and damage and deactivating the nuclear factor-kappa B (NF-κB) pathway [31]. During cognitive impairment, inflammation alters the microenvironment in the brain, which leads to alterations in glial and neuronal cells, and anti-inflammation partially restores neurogenesis, which attenuates cognitive impairment.

Chemotherapy-induced encephalopathy is a potential long-term side effect during cancer treatment [32] for which there is no effective treatment. *Epimedium*, as functional food, has many beneficial functions for human health. Therefore, we reviewed recent advances in natural compounds extracted from herbs and found Icariin (ICA) from *Epimedium* brevicornu Maxim. It possesses neuroprotective (e.g., excitotoxicity, oxidative stress, apoptosis, inflammation, and autophagy) and neurorestorative (e.g., angiogenesis, neurogenesis, and axon sprouting) biologic activity [33]. Studies have shown that ICA can play a regulatory role in astrocytes through the IGF-1 receptor signaling pathway, reducing inflammatory response [34].

**Figure 1:**
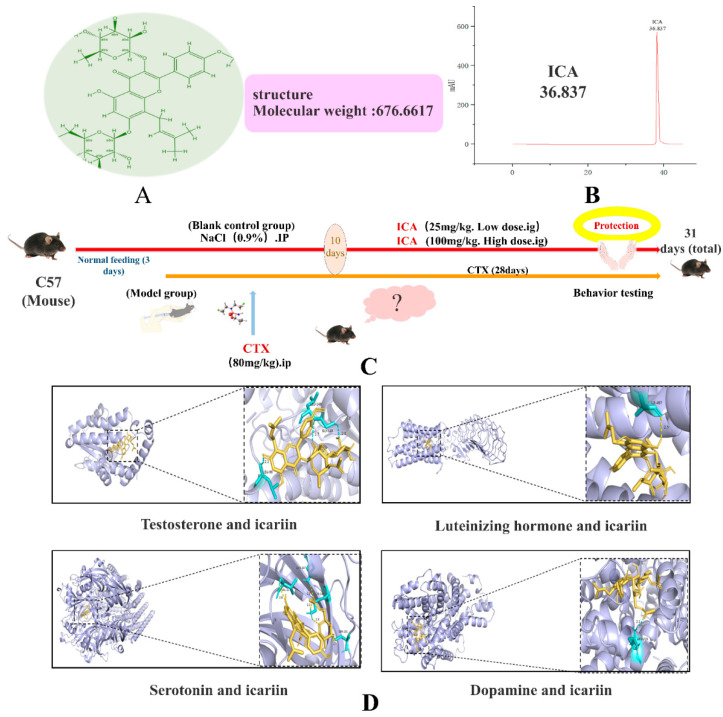
Research background and experimental process. (**A**) Icariin structure. (**B**) Icariin Atlas. (**C**) Experimental procedure. (**D**) Molecular docking results.

The structure of ICA is shown in Figure 1A, while the HPLC profile of ICA is shown in Figure 1B. The main active components of *Epimedium* are ICA, Baohuoside I (II), and Chaohuodin (A, B, and C), accounting for half of the flavonoid components [35]. The rich biological functions of ICA provide new ideas for the treatment of various brain injuries, and it has been found that ICA significantly improves the neurobehavioral function of traumatic brain injury (TBI) rats and attenuates pathological damage, brain tissue inflammatory factors COX-2, IL-1β, TNF-α, and the expression level of its regulatory proteins p-NF-κB-p65, p-ERK1/2, p-JNK, and p-p38 protein [36]. ICA pretreatment also promotes autophagy by activating the ERα and ERβ pathways, thereby reducing HIBD-induced apoptosis and exerting neuroprotective effects [37]. However, there are few studies on ICA in CTX-induced renal encephalopathy (RE).

In this study, by constructing an RE model, the neuroprotective effect of ICA under the regulation of serotonin and its beneficial effects on renal dysfunction were deeply explored. This study focused on the impact of ICA on neurodegenerative diseases via regulating the NF-κB inflammatory pathway, keap1-Nrf2 oxidative stress pathway, and apoptosis pathway. At the same time, we established in vitro models using HT22 and HEK293 cells to further verify the neuroprotective and renoprotective effects of ICA. This study provides an important experimental basis for the therapeutic potential of ICA in RE.

## 2. Results

### 2.1. Molecular Docking Results

Binding energies of less than −5.0 kcal/mol or −7.0 kcal/mol are generally considered to indicate that the conformation of the active compound and the main protein target exhibits strong and reliable binding interactions. The docking results of ICA with T, LH, 5-HT, and DA are −6.7 kcal/mol, −8.3 kcal/mol, −8.0 kcal/mol, and −6.8 kcal/mol. The results of molecular docking are shown in Figure 1.

### 2.2. In Vivo Results

**Figure 2:**
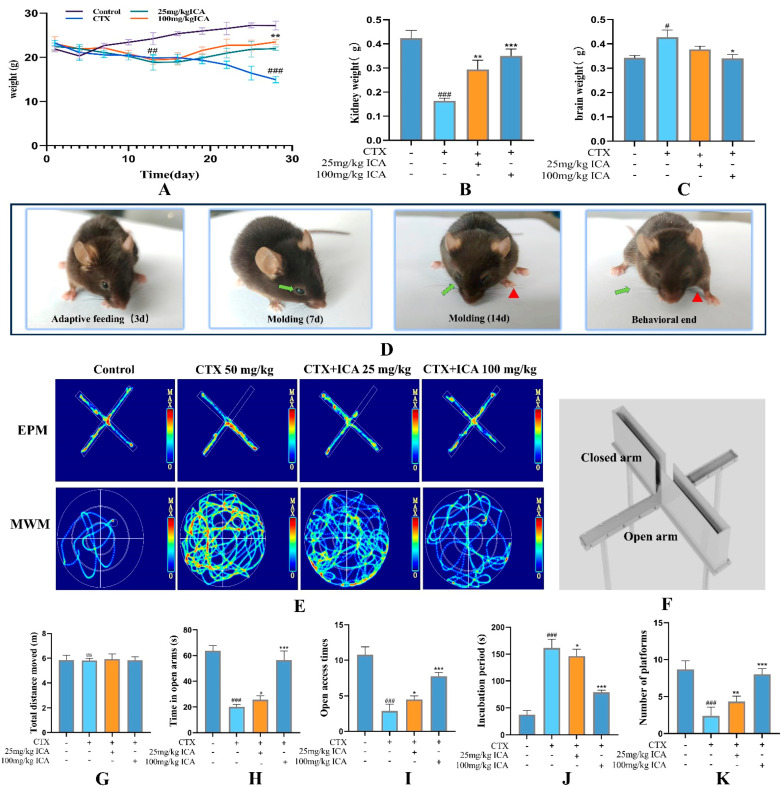
Behavioral changes induced by ICA on CTX in mice. (**A**) Mouse body weight change. (**B**) Mouse kidney weight. (**C**) Mouse brain tissue weight. (**D**) Comparison of mouse observations. (**E**) EPM and MWM. (**F**) EPM structure. (**G**) Total distance of EPM movement. (**H**) Time to enter open arm. (**I**) Number of times to enter open arm. (**J**) MWM latency. (**K**) Number of times MWM traversed the platform. *n* = 12, green arrow: eye disorientation; red triangle: limb paralysis. The CTX group compared to the control group with a difference (^#^ *p* < 0.05), a significant difference (^##^ *p* < 0.01), and a highly significant difference (^###^ *p* < 0.001); the administered group compared to the CTX group, with a difference (* *p* < 0.05), a significant difference (** *p* < 0.01), and a highly significant difference (*** *p* < 0.001), no significant difference (ns).

Changes in the body weight of mice (Figure 2A) showed that compared with the control group, the CTX group had already shown a decreasing trend in body weight at about day 13, and with the lengthening of the administration time of CTX and ICA, the comparison of the combination of the preliminary observations revealed that the weight loss of mice in the CTX group was accelerated. Compared with the control group, the kidney tissue weight in the CTX group decreased (Figure 2B), and the brain tissue weight increased (Figure 2C), with statistically significant differences (# *p* < 0.05 or ## *p* < 0.01). During CTX administration, mice showed reduced locomotion, frequent lethargy, dull eyes, gray fur, and slow limbs (Figure 2D). Compared with the CTX group, the body weight of the ICA group rose slowly after administration, and all the symptoms were reduced, which was significantly different (* *p* < 0.05 or ** *p* < 0.01). The increase was more evident in the 100 mg/kg ICA group compared with the 25 mg/kg ICA group, with a highly significant difference (*** *p* < 0.001). The above findings indicate that some nephrotoxicity, cerebral edema, and weight loss occurred after CTX administration; in contrast, all the symptoms were alleviated after ICA administration, which needs to be verified with an ELISA test, pathological analysis, and the protein blotting method.

#### 2.2.1. ICA Alleviates CTX-Induced Spatial Learning Memory in RE Mice

The behavioral trajectories of the mice in each group in the EPM experiment are shown in Figure 2E. There was no significant difference in the total movement distance (Figure 2G). Figure 2H,I show that, compared with the control group, the time for mice to enter the open arm and the number of mice entering the open arm in the CTX group were significantly lower, with a decrease of nearly 70%, which is highly significant (^###^ *p* < 0.001); compared with the CTX group, the time to enter the open arm and the number of mice entering the open arm in the two ICA groups were relatively increased (* *p* < 0.05, *** *p* < 0.001). This indicates that ICA could alleviate CTX-induced anxiety in mice.

The trajectories of the mice in each group of the MWM experiment are shown in Figure 2E. The results of analyzing the latency of the localization voyage, as well as the number of traversing platforms, are shown in Figure 2J,K. Compared with the control group, the latency of the CTX group significantly increased, and the number of traversing platforms was significantly reduced, both of which were highly significant (^###^ *p* < 0.001). The latency of both ICA groups decreased compared with that of the CTX group (* *p* < 0.05 or *** *p* < 0.001), and there was a relative increase in the number of plateaus crossed (** *p* < 0.01 or *** *p* < 0.001). This suggests that ICA can alleviate CTX-induced memory deficits in mice to a certain degree.

The results of the RMT experiment are shown in Table 1. Compared with the control group, the number of working memory errors and reference memory errors in the CTX group was higher than that in the control group, and the difference was extremely significant (^###^ *p* < 0.01). The number of working memory errors after administration was relatively reduced in the ICA group compared with the CTX group (** *p* < 0.01 or *** *p* < 0.001), and the number of reference memory errors was significantly reduced in the 100 mg/kg group compared with the 25 mg/kg ICA group (** *p* < 0.001). The number of reference memory errors was significantly reduced after 100 mg/kg ICA and 25 mg/kg ICA administration, and the difference was statistically significant in comparison (*** *p* < 0.001).

Behavioral tests suggested the success of the CTX-induced brain injury model, suggesting that ICA can alleviate aspects of learning, emotion, and memory in the CTX-induced impaired state of the mouse brain.

#### 2.2.2. Serum, Brain, and Kidney Tissue-Related Indicator Tests

**Figure 3:**
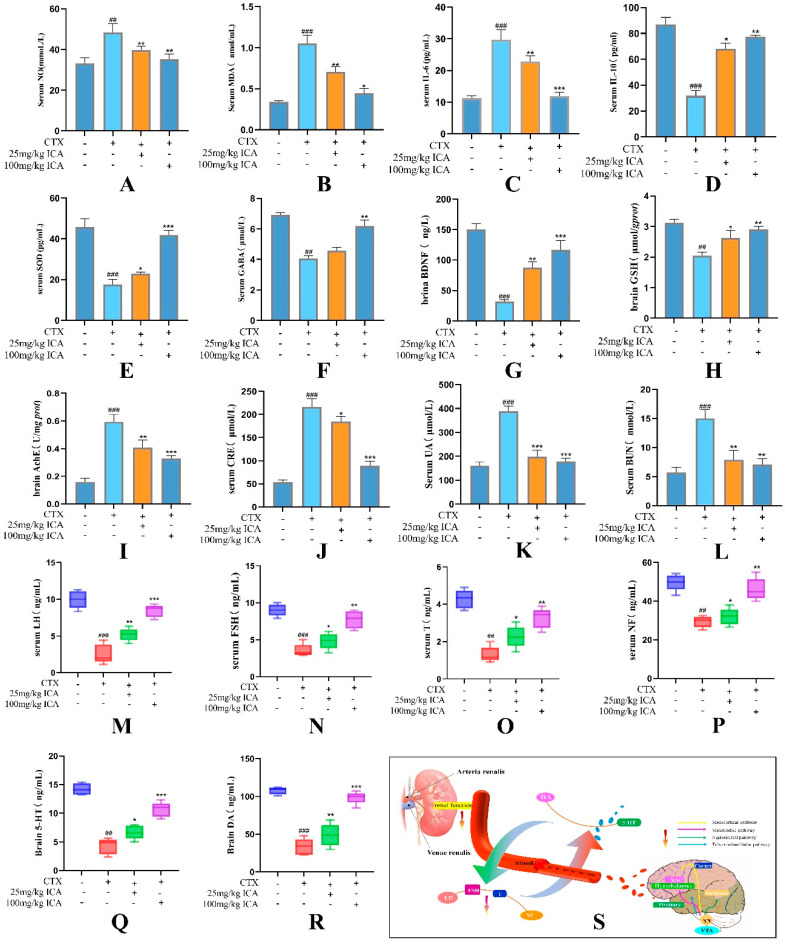
Results of serum and tissue biochemical indexes. (**A**) Serum NO level. (**B**) Serum MDA level. (**C**) Serum IL-6 level. (**D**) Serum IL-10 level. (**E**) Serum SOD level. (**F**) Serum GABA level. (**G**) Brain tissue BDNF level. (**H**) Brain tissue GSH level. (**I**) Brain tissue AChE level. (**J**) Serum Cre level. (**K**) Serum UA level. (**L**) Serum BUN level. (**M**) Serum LH levels. (**N**) Serum FSH levels. (**O**) Serum T levels. (**P**) Serum NF levels. (**Q**) Serum 5-HT levels. (**R**) Serum DA levels. (**S**) Kidney brain and serotonin. *n* = 3; the CTX group showed a significant difference (^##^ *p* < 0.01), and a highly significant difference (^###^ *p* < 0.001) compared to the control group; the administered group showed a difference (* *p* < 0.05), a significant difference (** *p* < 0.01), and a highly significant difference (*** *p* < 0.001) compared to the CTX group.

ELISA results analyzing serum revealed that compared with the control group, NO (Figure 3A), MDA (Figure 3B), and IL-6 (Figure 3C) were elevated to varying degrees, and IL-10 (Figure 3D), SOD (Figure 3E), and GABA (Figure 3F) were decreased in the CTX group, with all of them significant (^##^ *p* < 0.01 or ^####^ *p* < 0.001). Compared with the CTX group, the ICA group reversed this phenomenon after administration, reducing the levels of NO and MDAIL-6 while increasing the levels of IL-10, SOD, and GABA (* *p* < 0.05, ** *p* < 0.01, or *** *p* < 0.001). Compared with the two ICA groups, the 100 mg/kg ICA group had the best effect.

In order to further detect the degree of CTX-induced brain damage, brain tissue BDNF- (Figure 3G), GSH- (Figure 3H), and acetylcholinesterase (AChE)-related indexes (Figure 3I) continued to be examined. The results revealed that BDNF and GSH decreased (^##^ *p* < 0.01 or ^###^ *p* < 0.001). AChE was extremely significantly increased (^###^ *p* < 0.001) in the CTX group compared with the control group, and CTX inhibited the survival and differentiation of neuronal populations. This phenomenon was significantly reversed by ICA administration (** *p* < 0.01 or *** *p* < 0.001), which showed effects on the mouse hippocampal synaptic density and learning and memory abilities, with supportive effects.

CRE, BUN, and UA are the most commonly used biological indicators to respond to renal function, and a decrease in the glomerular filtration rate resulted in their elevation. As shown in Figure 3J–L, CRE, UA, and BUN in the serum of CTX-treated mice increased significantly (^###^ *p* < 0.001), indicating that CTX induced severe inflammation and oxidative stress in mice, resulting in renal histopathological damage and inducing renal dysfunction, and the nephrotoxicity model was successfully constructed. The changes in these indexes were reversed to varying degrees by the administration of ICA (** *p* < 0.01 or *** *p* < 0.001), indicating that ICA alleviated CTX-induced nephrotoxicity and improved renal injury, with the best effect observed in the 100 mg/kg ICA group (*** *p* < 0.001). Compared with the control group, the serum levels of LH, FSH, T, 5-HT, and DA in the CTX group were decreased (** *p* < 0.01 or *** *p* < 0.001). Compared with the CTX group, the ICA group had higher levels of all indexes (** *p* < 0.01 or *** *p* < 0.001) (Figure 3M–R).

Inflammatory factors and oxidative stress play crucial roles in brain and kidney injury. The above results demonstrate that ICA can alleviate CTX-induced RE by reducing oxidative stress, alleviating abnormal choline function, and inhibiting inflammatory factors.

#### 2.2.3. H&E, PAS, and Nissl Stain Results

**Figure 4:**
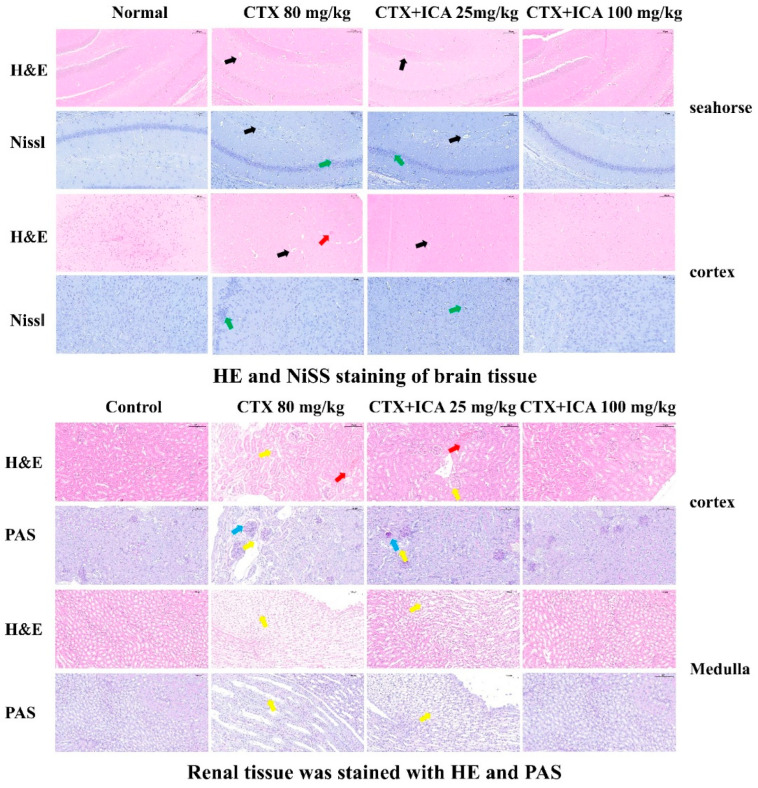
HE and Nissl in the cortex and hippocampus of brain tissue; HE and PAS in the cortex and medulla of renal tissue. Black arrows (vacuoles); green arrows (loss of Nissl and nuclear atrophy); yellow arrows (cellular vacuolization); blue arrows (glycogen deposition); inflammatory infiltrate (red arrows). Scale: 100 μm.

The HE staining of the brain in Figure 4 shows that the brain tissue of mice in the control group was structurally normal in all areas, with total neuronal cells, uniform cytoplasmic distribution, a light pink color, clear nuclear boundaries, intact morphology and structure of cortical cells, and dense gaps without edema. The neuronal cells in the dentate gyrus of the mouse hippocampus were rounded in shape, neatly arranged, and structurally clear and complete. Compared with the control, the brain tissue of CTX mice showed cortical edema, a significant decrease in the number of neuronal cells, an enlarged gap around neuronal cells and glial cells, and an increased number of vacuoles in the mesenchyme, with a significant difference (^#^ *p* < 0.05). The nuclei of the neuronal cells in the dentate gyrus of the hippocampus showed noticeable nuclear condensation and deep staining, with a significant increase in extracellular gaps, and the neurons of the hippocampal region were disorganized and loose, with an irregular arrangement, blurred nuclei, and apparent injury, showing significant differences (^##^ *p* < 0.01). Compared with the CTX group, the neuronal damage in the hippocampus of mice in the ICA group was significantly improved. The cortical cells were neatly arranged, the nuclei were more evident, and the difference was significant (*p < 0.05 or *** *p* < 0.001). The improvement effect was even more pronounced in the 100 mg/kg ICA group (*** *p* < 0.001), indicating that ICA significantly affected the pathologic damage of RE induced by CTX. The results showed that ICA had a significant improvement effect on CTX-induced NBI pathology.

Nissl is used to reflect the damage of nerve cells through the change in the number of Nissl vesicles and the morphology of neurons. The cortex structure of control group mice was found to be typical, the cell morphology was intact, the number of Nissl was typical, and clear, and the complete cytoplasm could be seen. Compared with control, the presence of multiple neurons in the CTX group was damaged, as evidenced by the presence of irregular neuronal cell bodies, increased vacuoles in the cortical interstitium, shrunken and discolored nuclei, and dry cytoplasm with vesicles, with highly significant differences (^###^ *p* < 0.001). Moreover, there were fewer, irregularly and sparsely arranged, and solidly constricted Nysted vesicles. Compared with the CTX group, the ICA group had less neuronal damage, neurons located in the center of the torso, normal structure of the regions, and intact morphology and structure of nerve cells. The 100 mg/kg ICA group tended to be more normal compared to the 25 mg/kg ICA group, and the difference was highly significant (*** *p* < 0.001). The experimental results indicated that ICA could alleviate CTX-induced cell damage in the cerebral cortex, as well as nidus loss and nuclear atrophy in hippocampal neurons.

The HE staining in Figure 4 shows that the renal tissues of mice in the control group had a standard structure in all areas, clear demarcation between the medulla and cortex, intact cellular morphology, and normal glomeruli with no lesions. Compared with the control group, the kidneys of the CTX group showed more morphological damage, including necrosis, inflammatory cell infiltration, the formation of casts, and swollen vacuolization of the nuclei of a large number of renal tubular epithelial cells, and the difference was significant (^##^ *p* < 0.01). Compared with the CTX group, the medullary and cortical structures in the ICA group were relieved and demarcated, fewer tubular epithelial cells were lysed, and fewer renal tubular lumens were seen with inflammatory cell infiltration, which was differential (** *p* < 0.05); the 100 mg/kg ICA group was almost normalized.

PAS staining of renal tissues revealed intact glomerular morphology without neutrophil infiltration, clear delineation of the medulla and cortex, well-defined demarcation, and no glycogen deposition in the control group. Compared with the control group, the renal tissues of mice in the CTX group had poorly demarcated medulla and cortex. The renal tubular dilatation and edema, glycogen deposition, inflammatory cell infiltration, and vacuolization were severe, and the difference was significant (^##^ *p* < 0.01). Compared with the CTX group, fewer renal tubular epithelial cells were lysed in the ICA group, less inflammatory cell infiltration was seen in the lumen of the renal vesicles, and a small amount of renal tubular dilatation and edema, and less glycogen deposition was seen in the ICA group. The difference was significant (* *p* < 0.05 or ** *p* < 0.01). The effect of the 100 mg/kg ICA group was even better, and no apparent inflammation was seen. The results showed that ICA could alleviate CTX-induced renal cortical and medullary cell injury, inflammatory infiltration, and glycogen deposition.

#### 2.2.4. Western Protein Blotting Results for Brain and Kidney Tissues

**Figure 5:**
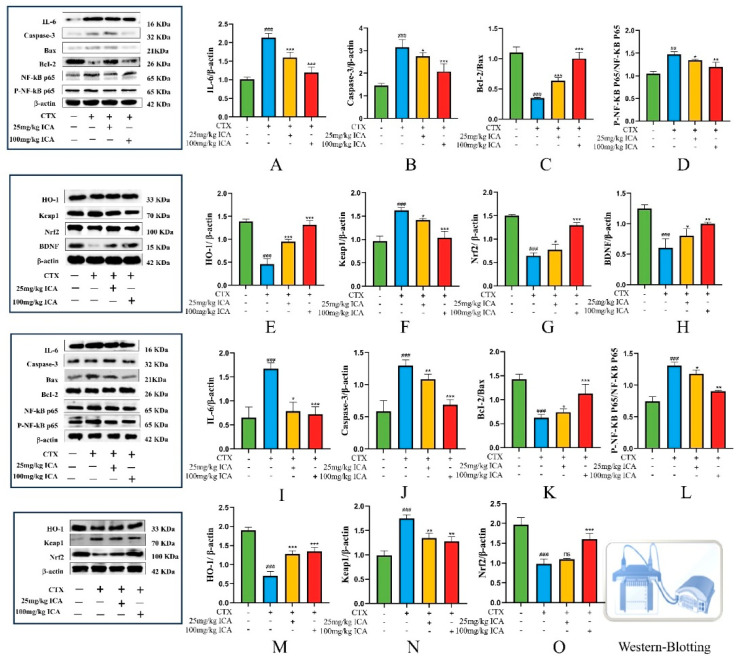
NF-κB, Nrf2-Keap1, apoptosis, and BDNF-related protein expression in mouse brain and kidney tissues. (**A**) Brain IL-6 protein expression. (**B**) Brain caspase-3 protein expression. (**C**) Brain Bcl-2/Bax ratio. (**D**) Brain P-NF-KB P65/NF-KB P65 ratio. (**E**) Brain HO-1 protein expression. (**F**) Brain Keap1 protein expression. (**G**) Brain Nrf2 protein expression. (**H**) Brain BDNF protein expression. (**I**) Kidney IL-6 protein expression. (**J**) Kidney caspase-3 protein expression. (**K**) Kidney Bcl-2/Bax ratio. (**L**) Kidney P-NF-KB P65/NF-KB P65 ratio. (**M**) Kidney HO-1 protein expression. (**N**) Kidney Keap1 protein expression. (**O**) Brain Nrf2 protein expression. *n* = 3. The CTX group showed a significant difference (^##^ *p* < 0.01), and highly significant difference (^###^ *p* < 0.001) compared to the control group; the administered group showed a difference (* *p* < 0.05), significant difference (** *p* < 0.01), and highly significant difference (*** *p* < 0.001) compared with the CTX group, no significant difference (ns).

In order to investigate the protective effects of ICA against NF-κB, Nrf2-Keap1, and apoptosis in the brain and kidney tissues of RE mice, the expression of related proteins in the brain and kidney of mice in each group was detected using Western blotting. The results showed that CTX treatment significantly improved IL-6 protein expression (Figure 5A,I), caspase-3 (Figure 5B,J), Keap1 (Figure 5F,N), and p65 (Figure 5D,L) in the brain and kidney compared with the control group and down-regulated the expression level of Bcl-2/Bax (Figure 5C,K), HO-1 (Figure 5E,M), Nrf2 (Figure 5G,O), and BDNF (Figure 5H), with significant differences (^##^ *p* < 0.01 or ^###^ *p* < 0.001). In contrast, ICA administration reversed the expression trend of these proteins to varying degrees (* *p* < 0.05, ** *p* < 0.01 or *** *p* < 0.001). Activation of the NF-κB pathway has been shown to regulate the Nrf2 pathway, possibly through the protein interaction between p65 and Keap1.

The present study suggests that ICA may promote the expression of anti-apoptosis-related proteins, inhibit apoptosis, reduce inflammatory factors, and regulate oxidative stress by regulating NF-κB, Nrf2-Keap1, and apoptosis-related pathways, thereby protecting against CTX-induced RE.

### 2.3. In Vitro Results

#### 2.3.1. Effect of ICA on CTX Injury to HT22 and HEK293 Cell Viability

It has been shown that ICA can provide new ideas in treating AD by mediating HT22 cells with the help of oxidative stress [38]. The results of using CCK-8 showed that the effective modeling concentrations of HT22 and HEK293 cells treated with CTX were 4 μM and 8 μM, respectively, and the low and high dosages of ICA were 5 μM and 20 μM and 10 μM and 40 μM, respectively; these were used as the optimal dosages for administration in later experiments.

**Figure 6:**
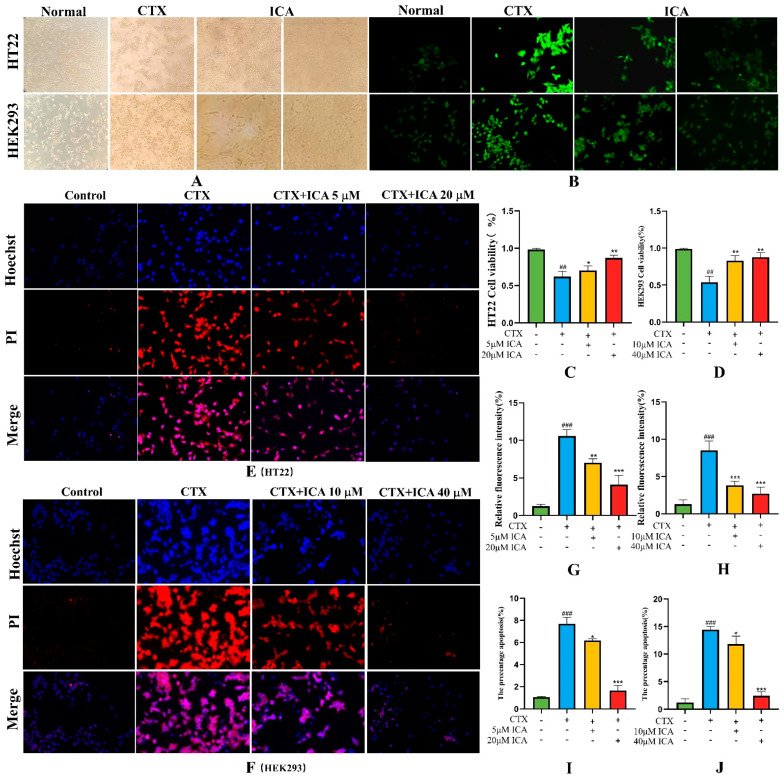
ROS and Hoechst 33342/PI assay results of cells. (**A**) Microscopic image pictures of HT22 and HEK293 cells during drug administration. (**B**) ROS fluorescence of cells. (**C**) Effect of ICA on viability of CTX-injured HT22 cells. (**D**) Effect of ICA on viability of CTX-injured HEK293 cells. (**G**) Intensity of ROS fluorescence of HT22 cells. (**H**) Intensity of ROS fluorescence of HEK293 cells. (**E**) HT22 cells Hoechst 33342/PI double staining. (**F**) HEK293 cell Hoechst 33342/PI double staining. (**I**) HT22 cell apoptosis percentage. (**J**) HEK293 cell apoptosis percentage. Fluorescence magnification: 20. The CTX group showed a significant difference (^##^ *p* < 0.01), and a highly significant difference (^###^ *p* < 0.001) compared to the control group; the administered group showed a difference (* *p* < 0.05), a significant difference (** *p* < 0.01), and a highly significant difference (*** *p* < 0.001) compared to the CTX group.

As shown in Figure 6A, it was found that the morphology and density of HT22 and HEK293 cells in the control group were intact and normal after spreading. The density of the cells was reduced, and cells appeared to float after CTX modeling. Compared with the CTX group, the floating was reduced and growth slowed after ICA administration, and the HT22 and HEK293 cells tended to be normal at 20 μM and 40 μM ICA doses as detected by CCK-8 (Figure 6C,D), which was significantly different (** *p* < 0.01 or *** *p* < 0.001).

#### 2.3.2. Intracellular ROS Detection

Using the properties of ROS, non-fluorescent probe molecules can oxidize to produce fluorescent products. By measuring the intensity of the fluorescence signal, the level of ROS in the cell can be indirectly known. Figure 6B,G,H show that CTX-treated HT22 and HEK293 groups had enhanced fluorescence intensity and increased ROS content, which was significantly different from the control group (^#^ *p* < 0.05). Compared with the CTX group, ICA administration suppressed a high level of intracellular ROS, and the fluorescence intensity gradually decreased, with the most apparent effect for high dose ICA (*** *p* < 0.001). The results indicated that ICA could improve ROS production and oxidative stress levels in CTX-induced HT22 and HEK293 cells.

#### 2.3.3. Effect of ICA on CTX-Induced Apoptosis

The Hoechst 33342/PI double-staining method was used to detect the degree of apoptosis. Figure 6E,F,I,J show that HT22 and HEK293 cells in the control group had the lowest blue light and almost no red fluorescence, and the blue fluorescence became brighter with CTX induction, along with a large number of red fluorescent spots and increased apoptosis rate (^#^ *p* < 0.05). After administration of ICA pretreatment, the brightness of blue and red fluorescence decreased sequentially, and the area of red fluorescence also decreased dramatically (** *p* < 0.01 or *** *p* < 0.001).

**Figure 7:**
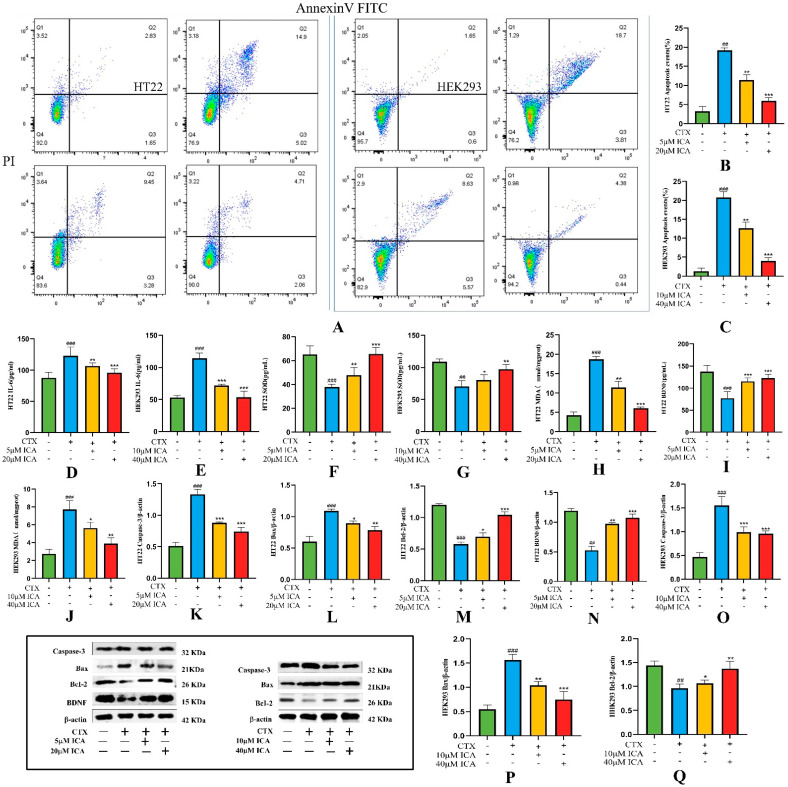
Annexin V-FITC/PI and apoptosis protein assay results. (**A**) Results of apoptosis detection by Annexin V-FITC/PI flow cytometry. (**B**) Apoptosis of HT22 cells. (**C**) Apoptosis of HEK293 cells. (**D**) IL-6 level of HT22 cells. (**E**) IL-6 level of HEK293 cells. (**F**) SOD level of HT22 cells. (**G**) SOD level of HEK293 cells. (**H**) MDA level of HT22 cells. (**I**) HT22 cell BDNF level. (**J**) HEK293 cell MDA level. (**K**) HT22 cell caspase-3 protein expression. (**L**) HT22 cell Bax protein expression. (**M**) HT22 cell Bcl-2 protein expression. (**N**) HT22 cell BDNF protein expression. (**O**) HEK293 cell cleaved caspase- 3 protein expression. (**P**) HEK293 cell Bax protein expression. (**Q**) HEK293 cell Bcl-2 protein expression. The CTX group showed a significant difference (^##^ *p* < 0.01), and a highly significant difference (^###^ *p* < 0.001) compared to the control group; the administered group showed a difference (* *p* < 0.05), a significant difference (** *p* <0.01), and a highly significant difference (*** *p* < 0.001) compared to the CTX group.

The results of apoptosis were detected by Annexin V-FITC/PI flow cytometry. As shown in Figure 7A–C, the total cell apoptosis rate was 4.48% and 2.25% in the control group of HT22 and HEK293 cells, respectively, and 19.92% and 22.51% in the CTX-induced group (^##^ *p* < 0.01 or ^###^ *p* < 0.001). The total cell apoptosis rate was decreased in the ICA-treated group (** *p* < 0.01 or *** *p* < 0.001), with 12.73% and 14.2% in the low-dose group and 6.77% and 4.82% in the high-dose group. It indicated that ICA could effectively inhibit CTX-induced apoptosis in HT22 and HEK293 cells.

#### 2.3.4. Detection of Biochemical Indicators

ICA treatment significantly inhibited the expression level of inflammatory factors and showed strong anti-inflammatory activity. CTX chemotherapy resulted in a sharp increase in IL-6 factor and MDA in inflammatory HT22 and HEK293 cells (Figure 7D,E,H,J), and Figure 7F,G,I show a decrease in SOD and BDNF (^##^ *p* < 0.01 or ^###^ *p* < 0.001), which was significantly reversed by ICA treatment (** *p* < 0.01 or *** *p* < 0.001), with a more pronounced effect of high-dose ICA. Correspondingly, ICA reversed these changes in a dose-dependent manner in CTX-induced RE. These results suggest that ICA can effectively inhibit inflammation and oxidative stress in CTX-induced RE.

#### 2.3.5. Western Protein Blotting Assay for Apoptosis-Related Proteins Caspase-3, Bax, Bcl-2, and BDNF Expression Levels

The Western blot results shown in the figure below indicate that the protein expression ratio of Bcl-2 and BDNF was significantly down-regulated after the induction of HT22 and HEK293 cells with μM CTX (Figure 7M,N,Q), while Figure 7K,L,O,P show a significant increase in the expression of caspase-3 and Bax proteins (^###^ *p* < 0.01 or ^####^ *p* < 0.001). When ICA was administered, the protein expression ratios of BDNF and Bcl-2/β-actin were significantly increased. In contrast, caspase-3 and Bax protein expression were significantly down-regulated compared with that in the CTX group (** *p* < 0.01 or *** *p* < 0.001). The WB results revealed that ICA may exert anti-apoptotic effects by regulating the expression of apoptotic pathway-related proteins. The results were consistent with those of in vivo studies.

## 3. Discussion

Studies have shown a link between kidney and brain dysfunction in common complications, including acute kidney injury (AKI) and cerebral P. falciparum infection [39]. There is significant interaction between the kidneys and brain in terms of physiological structure and functional regulation, which is mainly reflected in neuroendocrine regulation, metabolite exchange, and hemodynamic regulation, and cognitive deficits are prevalent in different patients with chronic kidney disease (CKD), which may lead to depression, reduced quality of life, and increased mortality. In conclusion, the interactions between the kidney and the brain are complex and multifaceted, and often, renal dysfunction increases cognitive abnormalities. 5-HT is the key to neuronal information transmission, and studies have shown that Xiaobupleurum decoction can reduce depression-like behavior in mice by enhancing the expression of hypothalamus and serum sex hormones to restore 5-HT and related hormone levels [40]. Serotonin is transferred from the synaptic gap back to the presynaptic neurons, thereby improving memory and cognitive impairment. Serum acts on many organs in the body, directly affecting the immune system, nervous system, growth, and development. The decrease in 5-HT and DA will decrease the regulation of LH, FSH, and T secretion. Changes in the levels of brain neurotransmitters, such as DA and 5-HT, indicate an imbalance of monoamine transmitters in the brain, which leads to brain disease [41], ICA can not only improve the reproductive ability and fertility of male animals, but also regulate serotonin, LH, and testosterone levels [42]. Molecular docking tests showed that ICA had good docking potential with serotonin and further linked the kidney and brain through the blood. Our study investigates the ameliorating impact of ICA on CTX-induced RE in terms of inflammation, oxidative stress, and apoptosis, emphasizing further exploration of the connection between the kidney and the brain at the serotonin level. T is a steroid hormone secreted by the ovaries and FSH stimulates the maturation and growth of follicles. Taking ICA for 30 days significantly increases T, reduces the levels of FSH and LH, and restores the disturbed hormones to normal levels.

**Figure 8:**
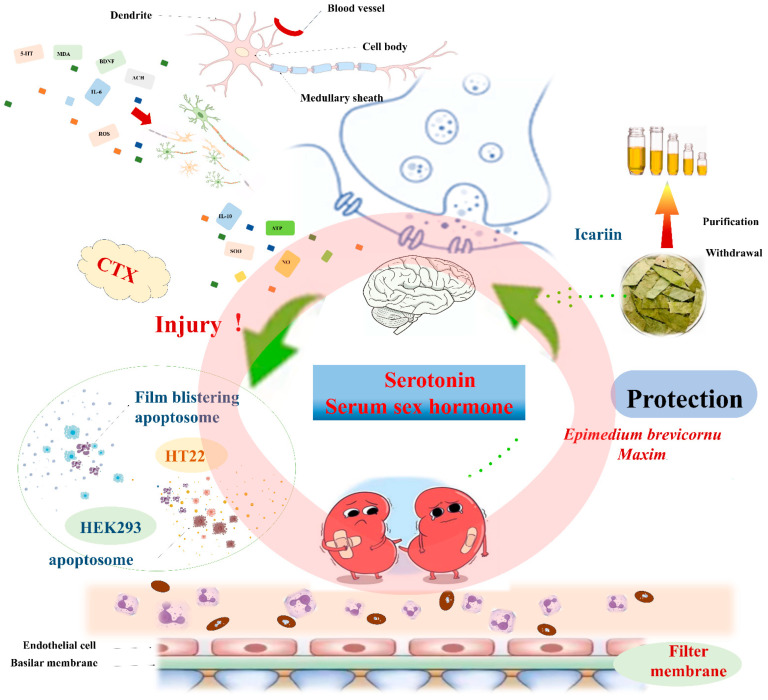
A framework of the Research and Discussion Sections. The kidney and brain are closely related, which is mainly reflected in the fact that kidney function directly affects the physiological function of the brain.

CTX is widely used in cancer, as well as in chemotherapy for autoimmune and immune-mediated diseases. In recent years, there has been a significant increase in the variety of renal and cognitive side effects caused by CTX therapy with severe nephrotoxicity, leading to the apoptosis and necrosis of renal tubular epithelial cells, the release of inflammatory factors, and the mediation of inflammatory responses [43]. However, this drug accelerates cognitive aging in cancer patients [44]. CTX can reduce synaptic plasticity by hindering the expression of BDNF, the phosphorylated cyclase response element-binding protein, synaptophysin, and the postsynaptic density protein 95 [45], exacerbating RE. New natural drugs or therapeutic agents are essential (Figure 8).

Many compounds used in cancer therapy are also isolated from natural herbs, and ICA is one of them. It has been extensively studied for its anti-inflammatory [36], antioxidant [46], and antidepressant activities [47], as well as for its therapeutic potential in the treatment of kidney injury [48] and brain injury [37,49].

Oxidative stress is one of the significant mechanisms of cell death in a variety of diseases and injuries and is closely associated with cognitive dysfunction. The excessive production of ROS is accompanied by a reduction in the capacity of the natural antioxidant system, which in turn leads to lipid peroxidation and increased MDA levels, the content of which can reflect the extent to which the cells of the body are subjected to free radical attack. Current biocatalytic production faces challenges, including low conversion rates and limited enzyme activity. Recent studies have shown that ICA can be produced from the leaf extract of *Epimedium* by a one-pot cascade of multifunctional glycosidase and rhamnosidase [50]. The pharmacodynamic mechanism of total flavones and Icariin of *Epimedium* has been studied extensively through various cell models and animal models at home and abroad. However, there are still some new challenges in neuroprotection research, especially clinical trials.

ICA treatment in our study significantly reduced CTX-induced serum and cellular MDA levels in mice, and the results suggest that ICA can alleviate CTX-induced RE and apoptosis by exerting its antioxidant function. Corresponding existing studies have shown that ICA can exhibit radioprotective effects against ionizing radiation-induced neurodegenerative disorders [51]. Combined with the Western blotting technique to detect Nrf2 and Keap1 proteins in the kidney and brain tissues, Keap1 protein expression was elevated in the kidney and brain tissues of mice in the CTX group compared with the control group, while the expression levels of Nrf2 and HO-1 proteins were reduced (^##^ *p* < 0.01 or ^###^ *p* < 0.001), which could have been reversed by ICA administration. It suggests that ICA can attenuate CTX-induced RE by regulating Keap1/Nrf2/HO-1 signaling pathway proteins.

NF-κB has long been recognized as a typical pro-inflammatory signaling pathway, and it has been found that CTX treatment activates inflammation in vivo, which subsequently triggers the activation of the NF-κB pathway, resulting in oxidative stress and apoptosis [11]. In the present study, we found that after the use of CTX, the inflammatory factor IL-6 in brain and kidney tissues surged compared with the control group, serum IL-10 expression decreased. ICA showed anti-inflammatory biological functions, and it was hypothesized that ICA might have a protective effect on CTX-induced RE and could play a role through the NF-κB signaling pathway. Western blotting results showed that, while the CTX group significantly up-regulated the ratio of the p-p65/p65 protein in mice kidneys and brains (^##^ *p* < 0.05 or ^###^ *p* < 0.001), the ICA group inhibited the phosphorylation of p65, thus confirming our conjecture that ICA may exert a protective effect against CTX-induced RE by inhibiting the NF-κB signaling pathway.

Through a series of behavioral experiments, as well as tissue H&E and Nissl and PAS staining pathology, it was found that the CTX group exhibited severe learning and memory deficits, as well as mood abnormalities, nucleus accumbent, neuronal damage, and deterioration of the cortex and hippocampus, which is in line with a previous study [52]. We speculate that spatial memory impairment and depressed mood may be related to the altered morphology and structure of neuronal cells, as well as apoptosis in the hippocampal region of its brain tissue. The activation of CNS glial cells, as well as the production of large amounts of cytokines and chemokines, the infiltration of peripheral immune cells, edema, and increased blood–brain barrier permeability, can lead to pathological neuroinflammation [53]. We determined the level of intracellular ROS, and the fluorescence results showed that the fluorescence intensity of CTX-induced cells was enhanced compared with that of the control group, and ICA treatment significantly inhibited the production of CTX-induced excessive intracellular levels of ROS, which was consistent with the results of the in vivo experiments.

The results of apoptosis detection using Hoechst 33342/PI, Annexin V-FITC/PI staining, and flow assay showed that the CTX group had significant apoptosis occurrence as high as 19.92% and 22.51%, respectively, and ICA could significantly inhibit the occurrence of apoptosis of HT22 and HEK293 cells induced by CTX. The HT22 and HEK293 cell Western blotting results showed that Bc1-2 protein expression was significantly down-regulated and Bax and caspase-3 protein expression was significantly up-regulated in the CTX group, and ICA effectively reversed this trend. In vivo experiments indicated that ICA may play a protective role by inhibiting apoptotic signaling pathways, which was consistent with the in vivo Western blotting results. This is consistent with a previous study [38] in which ICA inhibited BDNF expression in HT22 cells in a concentration-dependent manner, inhibited apoptosis, promoted SOD activity and the expression of GR, BDNF, and Bcl-2, and inhibited the expression of Bax and caspase-3. Meanwhile, our in vivo and in vitro studies also found that the BDNF pathway was involved in brain injury. Increased BDNF levels were found to help attenuate RE by Western blotting and ELISA, and this finding has been demonstrated in repeated transcranial magnetic stimulation to ameliorate cognitive deficits in radio encephalic mice by attenuating microglial cell pyroptosis and promoting neurogenesis through the BDNF pathway [54]. Previous studies have found that ICA preconditioning can reduce HIBD-induced apoptosis, which is consistent with the results of this study, and also indicates that ICA has a neuroprotective effect [37].

## 4. Methods and Materials

### 4.1. Molecular Docking

We tested the binding potential of ICA with LH, T, DA, and 5-HT via the molecular docking method, first identifying the objects that need molecular docking and then screening ICA through TCMSP. The ICA structure diagram was drawn in Chembiodraw Ultra 12.0 software, the protein molecule corresponding to the required target was searched in the UniProt database (accessed on 21 November 2024), the PDB format was downloaded, and the molecular docking software Auto Dock4.2.6 was used [55].

### 4.2. Drugs and Reagents

Icariin (Shanghai Macklin Biotech Co., Ltd. (Shanghai, China), purity ≥ 97.0%), cyclophosphamide (Shanghai Yuanye Biotechnology Co., Ltd. (Shanghai, China)).

Chromatography-grade methanol (Tianjin Shengxinyuan Weiye Trade Co., Ltd. (Tianjing, China)). SDS, Tris, 30% acrylamide, paraformaldehyde, glycine (Wuhan servicebio Biotechnology Co. (Wuhan, China)). Methanol, acetone, xylene, sulfosalicylic acid (Sinopharm Group Chemical reagent Co., Ltd. (Beijing, China). BCA protein quantification kit, CCK-8 (Lanjieke Science and Technology Co. (Beijing, China)). MDA (Item Number: S0131S), IL-6 (Item No.: PI328), BDNF (Code: PB070), Nitric oxide (NO) assay kit (Shanghai Biyuntian Biotechnology Co. (Shanghai, China)). SOD (Item Number: S0087), FSH, LH, T, DA, UA (Item No.: ADS-W-KY010), Cre (Item No.: ADS-W-FM034). 5-hydroxytryptamine (5-HT) assay kit (Hangzhou Pantechin Biotechnology Co. (Hangzhou, China)). Malondialdehyde (MDA) kit (Suzhou et al. Limited Biological Company). Primary and secondary antibodies (Wan Class Biotechnology Co., Ltd., Shenyang, China). Penicillin, Streptomycin, Fetal Bovine Serum (FBS), Trypsin, DMSO (Beijing Solebo Co., Ltd., Beijing, China).

### 4.3. Instrumentation

CO_2_ cell incubator, high-performance liquid chromatograph (Agilent 1260, Santa Clara, CA, USA), microscope camera system, multifunctional enzyme labeler (Thermo, Waltham, MA, USA), cryogenic high-speed centrifuge (Eppendorf, Hamburg, Germany), and ultrasonic cell crusher (Suzhou Sonic, Suzhou, China).

### 4.4. In Vivo Experiments

#### 4.4.1. Grouping and Processing of Animals

C57BL/6 male mice (5–6 w) weighing about 20–25 g were purchased from Changchun Yisi Laboratory Animal Technology Co., LTD. (Changchun, China). Mice were fed at 22–25 °C, relative humidity was 45–60%, 14 h light was alternated with 10 h darkness, and mice were free to eat and drink. The Experimental Animal Welfare and Ethics Committee of Jilin Agricultural University determined that the research project complies with ethical standards for experimental animals. The ethics review acceptance number is 20211011003. (Experimental Animal Center, Jilin Agricultural University License: SYXK (ji) 2018–0023). In the present experiment, mice were divided into four groups (*n* = 12), and changes in water intake, hair, and body weight were observed. The specific treatments were as follows:

Control group: daily intraperitoneal injection of an equal volume of saline for 28 days; regular feed and water.

CTX group: 80mg/kg intraperitoneal injection, 28 days.

CTX + ICA high-dose group: 100mg/kg ICA by gavage on the seventh day, 80mg/kg CTX by intraperitoneal injection one hour later.

CTX + ICA low-dose group: 25mg/kg ICA by gavage on day 7, 80mg/kg CTX by intraperitoneal injection one hour later.

As shown in Figure 1C, kidney, brain, serum, and other samples were taken 24h after the final treatment for subsequent experiments.

#### 4.4.2. Behavioral Tests

1. Elevated plus maze, EPM

The EPM device assessed whether the experimental animals were anxious by observing their exploratory behaviors on the open arm and avoidance behaviors on the closed arm [56]. The ambivalence of rats and mice was used to assess their response to potential threats and unsafe areas by collecting the duration of open-arm residence and the number or percentage of entries. The higher the percentage, the lower the anxiety. The mice were placed in the junction area of the open and closed arms, we clicked “Start”, and their trajectories on the elevated maze were automatically recorded within 3 min. The total distance, the number of times the mice entered the open arm, and the time of movement in the elevated cross mazes were analyzed.

2. Morris water maze, MWM

The MWM comprises localization navigation and space exploration [57]. The amount of time the animal spent in the target quadrant (the quadrant where the platform was originally placed) and the number of times it entered that quadrant were used as indicators of spatial memory. It consists of a circular pool with a diameter of 120 cm, divided into one, two, three, and four quadrants. The experimental time was set to 300 s. If the mice did not find the platform’s position, the success latency was defaulted to 300 s. The mice were placed on the platform for 10 s to learn to adapt, and on the last day, the platform was removed, and the number of times they crossed the platform and the time of the first time they crossed the platform within 300 s was recorded, thus assessing the mice’s memory ability.

3. Radial arm maze test, RMT

The RMT consists of 8 radiating arms and an octagonal region. The mice were fasted for 24 h at the beginning of training, with unlimited water intake, and trained for five days. Only baits were placed in arms 1 and 6, and after 15 s of acclimatization, the door of each arm was opened, and the mice were free to forage for food until they finished foraging for all the arms for 3 min or less. Working memory error: mice entered the food arm again in the same test. Reference memory error: mice entered the arm without food.

4. Testing serum and brain and kidney tissue (biochemical indicators)

After the behavioral study, the mice were weighed and recorded, and the cervical vertebrae were removed after blood was taken from the eyeballs. At 4 °C, centrifuged at 2000 r/min for 15 min, and the supernatant was taken and stored it at −80 °C (high-speed refrigerated centrifuge: Thermo Fisher Technology Co., Ltd.). Cerebral and renal tissue was collected, weighed and immobilized. NO, MDA, IL6, IL-10, SOD, GABA, BDNF, FSH, LH, T, DA, 5-HT, GSH, AChE, Cre, UA and BUN were determined strictly according to the kit instructions.

5. Histopathological observations

Hematoxylin and eosin (HE) staining is commonly used for histopathological examination [58], Nissl staining is used for observation of nidus in the hippocampal region of mice [59], and PAS staining is used for observation of renal pathological damage [60]. Parts of the brain and kidney tissues were fixed with 4% PFA—dehydration [61], transparency, embedding, sectioning, slide processing, HE, PAS, and Nissl staining.

6. Western blotting

Brain and kidney tissues were removed, and 1 mL of lysis solution (PMSF was added before use at a working concentration of 1%) was added to each 1 g of tissue in an ice bath environment to grind them. After the tissue was completely dissolved by Ripa protein lysis ultrasound, they were centrifuged at 4 °C at a speed of 12,000 r/min, and the protein concentration was determined. The same amount of protein was isolated by SDS-polyacrylamide gel electrophoresis and transferred to a polyvinylidene fluoride (PVDF) membrane. The membrane was blocked with 5% (*w*/*v*) skim milk in Tris-buffered saltwater containing 0.05% Tween 20 (TBST) under constant shaking. A certain proportion of antibodies was incubated for 12 h, the membrane was washed 3 times for 10 min each time, (each time with TBST), and then the anti-rabbit secondary antibodies bound with horseradish peroxidase were incubated at room temperature for 1 h. Incubation was performed with primary antibodies which were all purchased from Shenyang Wanlei Biotechnology Co., Ltd. (Shenyang, China): IL-6 (1:1600), p65 (1:1600), p-p65 (1:1000), Caspase-3 (1:800), Bax (1:800), Bcl-2 (1:800), Keap1 (1:1600), Nrf2 (1: 800), HO-1 (1:1600), BDNF (1:1600),β-actin (1:800), and secondary antibody (1:8000). ECL developer solution chemiluminescence was developed, and the expression of each protein was analyzed using Image J software (version 1.48v, National Institutes of Health, Bethesda, MD, USA).

### 4.5. In Vitro Experiments

The HT22 cell line is a mouse hippocampal neuron cell line, and HT22 cells are mainly used in the study of neurodegenerative diseases. HEK293 cells are human embryonic kidney cells which are more suitable for our study and have higher growth efficiency. The cell lines HT22 and HEK293 were from the research group’s pre-frozen storage (purchased from Jiangsu Kaiji Biotechnology Co., Ltd., Nanjing, China). HEK293 is a typical cell line derived from human embryonic kidney cells.

#### 4.5.1. Cell Recovery and Culture

We removed the frozen tubes of HT22 and HEK293 cell lines from the liquid nitrogen tank and thawed them by shaking them repeatedly for 1–2 min in a 37 °C water bath. We then added one times the volume of the complete culture medium to a 15 mL centrifuge tube and centrifuged it at 1200 r/min for 5 min (DMEM culture-medium purchased from Jiangsu Kaiji Biotechnology Co., Ltd.). We discarded the supernatant and added 4 mL of the complete culture medium and mixed it well. Lastly, we put it into the incubator at 37 °C containing 5% CO_2_ for incubation.

#### 4.5.2. Screening for Safe Drug Concentrations

When the growth density of HT22 and HEK293 cells reached 80–90%, the cell density was adjusted after digestion by pancreatic enzymes, and 100 μL cell fluid was added to each hole of a 96-well plate with a density of about 1 × 10^5^/well. The cells were incubated for 12 h and then starved for 12 h. Subsequently, CTX concentrations of 0.5 μM, 1 μM, 2 μM, 4 μM, 8 μM, 16 μM, 32 μM, and 64 μM were added to incubate the cells for 2 h and screened for LD50 values. Similarly, ICA concentrations of 1 μM, 5 μM, 10 μM, 20 μM, 40 μM, 80 μM, 120 μM, and 240 μM were set to screen the optimal administration of high and low doses, and six replicate wells were incubated for 12 h. The drug-containing medium was discarded, and the absorbance was measured at 450 nm by adding 1% serum medium, followed by CCK-8 [62]. Cell viability was calculated.

Cell viability = (OD experimental group − OD blank group)/(OD control group − OD blank group) × 100%

#### 4.5.3. Detection of Cell Viability by CCK-8 Assay

According to the screening results, we re-lay the plate to administer the treatment and performed starvation treatment for 12 h for all groups except the blank control group, according to the CTX modeling drug concentration to treat the cells for 12 h; we then performed CTX + ICA treatment for 12 h, discarded the drug-containing medium, added 1% serum medium, and then added CCK-8 at 450 nm to measure the absorbance. Cell viability was calculated [63].

#### 4.5.4. ROS Detection

A fluorescent probe was used to measure the intracellular ROS content (ROS No. G1706-100T, Xavier Biological Co., Ltd., Wormit, UK). DCFH-DA was hydrolyzed to DCFH when it entered the cells, and ROS then oxidized DCFH to produce DCF, which emitted green fluorescence. In this experiment, the cells were washed twice with PBS according to 2.4.3, 5 μM DCFH-DA was added, and the cells were incubated for 20 min at 37 °C in a cell culture incubator. They were then washed three times with PBS and observed under a fluorescence microscope. We diluted DCFH-DA in serum-free medium at a concentration of 10 micromol/L at 1:1000.

#### 4.5.5. Hoechst 33342/PI, Annexin V-FITC/PI Staining, and Flow-Through Detection of Apoptosis

Hoechst 33342 can be taken up by living cells and binds to DNA to fluoresce blue under UV light, and PI stains dead cells to produce red fluorescence [64]. After treatment, each well was stained with 5 μL of Hoechst 33342, 5 μL of PI, and 1 mL of cell staining buffer for 20 min, and the intensity of cell fluorescence was observed under a fluorescence microscope after washing with PBS.

Apoptosis was detected by Annexin V-FITC/PI flow cytometry. Cells undergoing early apoptosis were positive for Annexin V but negative for PI, while cells undergoing late apoptosis or necrosis would stain positive for Annexin V and PI.

#### 4.5.6. Detection of Biochemical Indicators

We extracted the cell culture medium after the operation in accordance with Section 4.5.3, and the relevant tests must be carried out in strict accordance with the kit instructions. The main detection indexes were MDA, IL-6, BDNF, and SOD.

#### 4.5.7. Cell Western Blotting

The apoptotic pathway caspase-3, Bcl-2, Bax protein expression, and BDNF protein was detected using protein immunoblotting.

### 4.6. Statistical Analyses

All data are expressed as the mean earth standard deviation. Statistical analysis was performed using SPSS 21.0 (SPSS et al., Chicago, IL, USA). Statistical differences were assessed using a two-tailed test or one-way analysis of variance (ANOVA), and *p* < 0.05 was considered statistically significant. Statistical plots were generated by GraphPad Prism 8.0.1 (GraphPad et al., San Diego, CA, USA).

## 5. Conclusions

In this study, we revealed the potential reno- and neuroprotective mechanism of ICA by constructing a CTX-induced RE model. The results of in vivo experiments indicate that ICA may play a protective role by regulating the NF-κB inflammatory signaling pathway and the Nrf2-Keap1 oxidative stress pathway. In addition, based on in vitro CTX-induced HT22 and HEK293 cell models, we found that ICA could significantly inhibit apoptosis and effectively ameliorate cell damage. This study not only elucidates the beneficial effect of ICA on CTX-induced RE but also provides an important theoretical basis and experimental support for the development of ICa-based treatment of renal encephalopathy.

## Figures and Tables

**Table 1:** Results of working memory errors and reference memory errors for the eight-arm maze.

Group	*n*	Dose	Error of Working Memory	Reference Memory Error
Control group	12	——	2.11 ± 0.23	3.42 ± 0.11
CTX	12	80 mg/kg	7.56 ± 0.33 ^###^	10.44 ± 0.31 ^###^
ICA	12	25 mg/kg	5.04 ± 0.29 **	7.11 ± 0.26 ***
ICA	12	100 mg/kg	3.48 ± 0.11 ***	5.11 ± 0.22 ***

*n* = 3. The CTX group showed a highly significant difference (^###^ *p* < 0.001) compared to the control group, and the administered group showed a significant difference (** *p* < 0.01), and highly significant difference (*** *p* < 0.001) compared with the CTX group.

## Data Availability

The authors acknowledge that data supporting the results of this study can be found in the article.

## Abbreviations

ICA	Icariin
CTX	cyclophosphamide
ROS	reactive oxygen species
BDNF	brain-derived neurotrophic factor
MDA	malondialdehyde
IL-6	Interleukin-6
NO	nitric oxide
SOD	superoxide dismutase
DMEM	Dulbecco’s modified eagle medium
Cre	creatinine
UA	uric acid
BUN	urea nitrogen
Nissl	Nissant staining
PAS	periodic acid–Schiff
HE	hematoxylin–eosin
FSH	follicle-stimulating hormone
LH	luteinizing hormone
T	testosterone
DA	dopamine
5-HT	serotonin
AD	Alzheimer’s disease
